# Primary ectopic substernal thyroid cancer with trachea relapse: a case report and opinions of management

**DOI:** 10.1186/s12957-016-0853-1

**Published:** 2016-03-31

**Authors:** Rui-Min Ma, Lin Lv, Shu-Rong Zheng, Jie You, Du-ping Huang, Gui-Long Guo

**Affiliations:** Department of Oncology, The First Affiliated Hospital of Wenzhou Medical University, Wenzhou, 325000 China

**Keywords:** Ectopic thyroid cancer, Substernal, Local invasion, Trachea relapse, Stent

## Abstract

**Background:**

Ectopic substernal thyroid is a rare symptom of thyroid disease that entirely results from the developmental defects at early stages of thyroid embryogenesis and during its descent. Cases were seldom reported as primary ectopic substernal thyroid cancer, especially those with severe local invasion and tracheal relapse.

**Case Presentation:**

In this report, the patient presented odynophagia and a sense of progressing swallowing obstruction. She underwent total thyroidectomy and lump resection. However, she refused to use postoperative radioactive iodine or take adjuvant external-beam radiotherapy, except for thyroid hormone replacement therapy. Tracheal relapse was observed after 6 months. Tracheal stent was used to reconstruct the airway twice.

**Conclusions:**

Trachea invasion might be a worse independent predictor of prognosis than any others and should be given particular attention. Furthermore, tracheal stent might be a palliative option for patients with tracheal relapse.

## Background

Thyroid cancer originates in thyroid tissue. It is common but not deadly [1]. The 20-year disease-free survival is about 90 % after operation [2]. Thyroid cancer can be easily identified through ultrasound test or physical examination. However, all optimistic results mentioned above are connected to eutopic thyroid cancers (>99 %) [2]. When thyroid cancer originates ectopically, it would be difficult to diagnose apace [3].

Ectopic thyroid is reported in approximately 1 per 100,000–300,000 individuals and entirely results from the developmental defects at early stages of thyroid embryogenesis and during its descent [4]. Occurrences in the substernum and mutation to cancer of ectopic thyroid are extremely rare. In this paper, we report a case of primary ectopic substernal thyroid cancer with severe local invasion.

## Case presentation

**Fig. 1:**
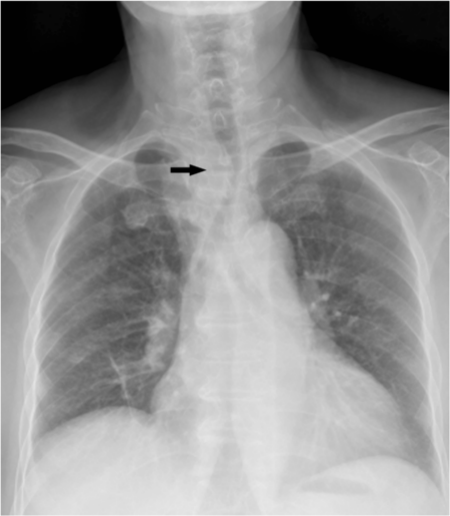
Chest radiograph: the trachea was shifted to the left, and there was mass or something faintly looming

**Fig. 2:**
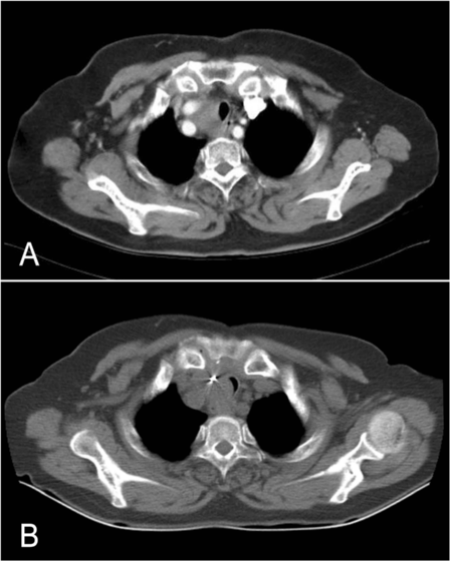
Computed tomography (CT). **a** Before surgery: enhanced CT scan noted a large substantive mass around the right common carotid artery bifurcation. **b** Six months after surgery: CT scan showed the trachea narrowed, in the level of the thoracic inlet, next to the silver clips

A 67-year-old woman was admitted with a 2-month medical history of odynophagia and a sense of progressing swallowing obstruction. She denied the other medical history, except for hypertension, which she had controlled well by pills. Her physical examination was normal, except for a tracheal shift. Doppler ultrasound on the neck revealed bilateral thyroid nodules and a substantive bump at the entrance of the thorax. Chest radiograph showed a tracheal shift (Fig. 1). Enhanced computed tomography (CT) scan of the neck noted a large substantive mass below the right lower pole of the thyroid with calcification, and two lobes of the thyroid both had nodules without any hint of lymph nodes (Fig. 2a). Core needle biopsy and immunohistochemistry revealed papillary thyroid cancer (PTC).

**Fig. 3:**
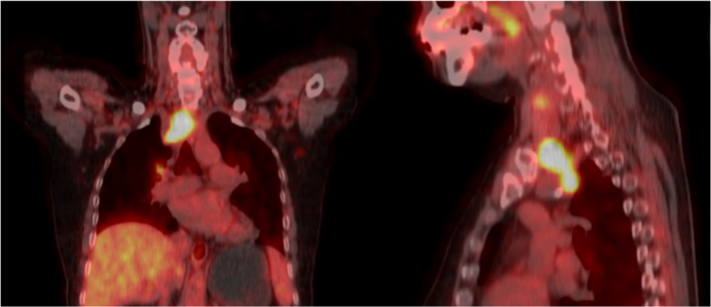
PET-CT revealed a high metabolic tumor under sternal bone

**Fig. 4:**
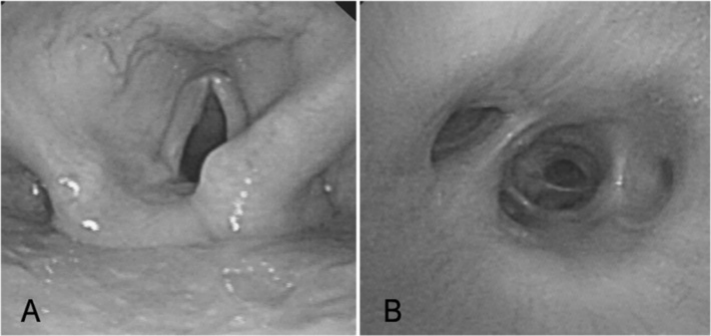
**a** Laryngoscopy showed an immobile vocal cord. **b** Bronchoscopy found nothing abnormal

The positron emission tomography-computed tomography (Fig. 3) indicated that the lump from the thoracic inlet area to upper mediastinum had high metabolism, and malignant neoplasm should be considered. Moreover, the tumor, along with surrounding invasion, caused the trachea to migrate to the left. However, the metabolic image of the mediastinal and hilar lymph nodes increased mildly, which was non-specific. Both sides of the thyroid might also favor nodular goiter. Laryngoscopy showed right vocal cord paralysis. The bronchoscopy and cytopathology of the brush fluid were normal (Fig. 4). The tumor mark and thyroid function tests were almost normal.

After all the examinations, we still had no idea whether the mass metastasized to the substernum or originated there. We opted to diagnose after excision. During operation, we found multiple nodules in both sides of the thyroid, as well as the mass, which was right behind the sternum, outside of the trachea and inside of the right carotid artery. The mass was an observed invasion of the trachea, carotid artery, and the recurrent laryngeal nerve. And, it was so conglutinated with the right carotid artery that it could not be removed completely. Thus, we firstly took total thyroidectomy with cervical lymph dissection of the right VI area. Subsequently, we used sternotomy to excise more of the lump after separating it from the local (right carotid artery, trachea, and recurrent laryngeal nerve) gradually; silver clips were left to mark the residual cavity in preparation for radiotherapy. Postoperative pathology confirmed that the lump was primary ectopic substernal papillary thyroid cancer, and the eutopic thyroid was nodular goiter. After surgery, the patient had an excellent recovery.

However, she refused to use postoperative radioactive iodine (RAI) or take adjuvant external-beam radiotherapy (EBRT), which had been planned before surgery. Instead, she took Euthyrox (100 mg every morning) for relapse prevention.

**Fig. 5:**
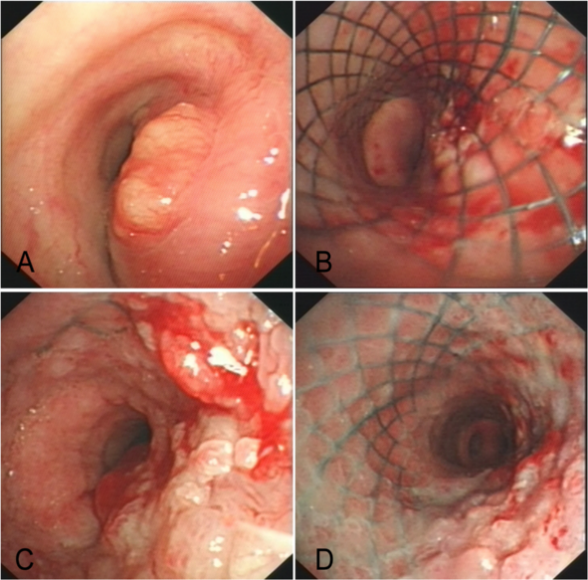
**a** Before stenting, the neoplasm narrowed nearly 70 % of the lower trachea. **b** After stenting, the airway was reconstructed for free respiration. **c** Before stenting, the neoplasm narrowed nearly 75 % of the airway at the second time just above the trachea carina. **d** After stenting, the airway was reconstructed for the second time

Six months later, she complained of dyspnea. CT showed that the trachea narrowed in the level of the thoracic inlet, next to the silver clips (Fig. 2b). Bronchoscopy confirmed that it was blocked by something new, and pathological diagnosis revealed that it came from thyroid cancer but was poorly differentiated. Tracheal stent was inserted via bronchoscopy to reconstruct the airway for free respiration (Fig. 5a, before stenting; Fig. 5b, after stenting). After another 6 months of using Euthyrox, a second tracheal stent was inserted because of recurring dyspnea (Fig. 5c, before stenting; Fig. 5d, after stenting).

### Discussion

After diagnosis and treatment of this woman, the following points were obtained.

#### Part. 1 Whether adjuvant RAI or EBRT should be taken?

A patient with PTC usually has an exciting 5-year overall survival rate (OS) of 97.5 % [5]. But, some risk factors for recurrence and death still exist. The most correlative factors affecting mortality are maximum tumor diameter (≥4), pre-operative neck gross metastasis, extrathyroidal invasion, and metastases [2, 6]. Postoperative RAI should generally be administered for these variants as they will generally be intermediate to advanced tumors [5]. In addition, old age ≥45, surrounding invasion, distant metastases, and gross locoregional residual are indicators to perform adjuvant EBRT [7]. EBRT will improve their survival and reduce the recurrent rate (51 % control group vs. 8 % EBRT group) [8]. The toxicity-related side effects of EBRT, such as skin reaction, esophagitis, and laryngeal irritation, are limited and resolved during follow-up [9].

In our case, the patient is a 57-year-old woman, and the tumor invaded the surroundings, such as trachea, right carotid artery, and right recurrent laryngeal nerve. It became even worse while the tumor cannot be completely excised because of the severe invasion in the right carotid artery. Depending on all of the above, RAI and EBRT are necessary for the patient just after the surgery.

However, tracheal relapse was poorly differentiated, and the RAI therapy had lost the opportunity to benefit.

#### Part. 2 Should we excise the invaded common carotid artery?

The excision of big arteries was taken into account when the tumor invaded the vessels so severely that it cannot be removed completely alone. Therefore, a better option should be selected between postoperative radiation therapy without excision of the carotid artery and replacement surgery of vessels.

The prognosis of those who have local invasion is worse than those who do not. Vessel invasion is a predictable index [10]. Nevertheless, many complications peri-operatively and postoperatively are present for this index, such as neurologic sequelae, graft infection or occlusion, and cerebral ischemia. Embolization and hemorrhage also cannot be ignored [11]. Hence, whether to reconstruct the invaded vessels or not is a dilemma.

We searched the PubMed database, but there are few literatures about the controversy, in addition to cases involving vessel reconstruction along with thyroid cancer. It is said that it is helpful to decrease patients’ long-term metastasis and locoregional recurrence with vascular invasion artery resection and reconstruction [12, 13].

However, other studies show that patients with T4 tumors can also have a good 5-year OS after adjuvant RAI and EBRT therapies [8]. Furthermore, locoregional failures all occur at the neck node area [9]. OS is not significantly different from those re-operated within cervical lymph node metastasis [14].

In our case, the common carotid artery was invaded, but no obvious tumor-induced blockage was observed (Fig. 3). Considering that the patient should take RAI and EBRT therapies, which depended on severe local invasion and old age (≥45), we opted for a milder surgery therapy after obtaining the family’s approval and consent.

Unfortunately, the patient refused the therapy after surgery. Nonetheless, the cervix condition was not too bad, and no distant metastasis was observed.

#### Part. 3 Should we excise the invaded trachea and reconstruct it?

Tracheal invasion, one third of the extrathyroidal extension, is an independent predictor of death, followed by hemoptysis and dyspnea, in approximately 6 % of thyroid cancer [15]. The status of trachea invasion can be divided into several stages. Stage A disease invades through the capsule of the thyroid gland and invades the external perichondrium of the trachea. Stage B disease invades between the rings of cartilage or causes minor cartilage destruction. Stage C disease invades through the cartilage or between the cartilaginous rings into the lamina propria of the tracheal mucosa or in the submucosal area. Stage D disease is a full-thickness invasion with the expansion of tracheal mucosa that is visible through a bronchoscope as a nodule or ulcerated mass [16]. Generally, the patients without residual have the greatest prognosis [17]. The patients with stage A invasion have an almost identical prognosis after shaving resection compared to segmental tracheal resection.

This woman with stage A trachea invasion in our case had shaving resection because of the granted adjuvant RAI and EBRT therapy. She refused the adjuvant therapy, and the gross relapsed through the trachea without having invaded the common carotid artery and other distant metastasis. Interestingly, the tumor invaded through the trachea faster than the artery, which had been invaded and had not been excised with all invasion tissues. Shaving resection may have destroyed the tracheal anatomy and made it easier to invade through than before. However, the survival period and disease-free survival period of having shaving resection, even the survival period of having palliative surgery, are much better than those without any airway operation in the patients of locally invaded thyroid cancer. Hence, trachea invasion might be a worse independent predictor of prognosis than any other local invasion. Therefore, aggressive procedures should be performed to protect the patient from relapsing. If tracheal relapse occurs, the stent might solve the respiration problem safely. Stent can be used a few times to release the patient’s dyspnea and fear.

## Conclusions

Primary ectopic substernal thyroid cancer is rare. When it happens, its local condition should be considered carefully. If the common carotid artery is involved, the adjuvant RAI and EBRT should be undertaken to get better OS. If the trachea is invaded, then aggressive surgical procedures should be considered, such as full-thickness window resection or segmental tracheal resection, instead of shaving resection. Tracheal stent might also be a palliative option for a patient who has tracheal relapse and cannot be removed.

### Consent

Written informed consent for the publication of this case report and all the accompanying images were obtained from the patient. A copy of the written consent is available for review by the Editor-in-Chief of this journal.

## Abbreviations

CT: computed tomography
EBRT: adjuvant external-beam radiotherapy
OS: overall survival
PET-CT: positron emission tomography-computed tomography
PTC: papillary thyroid cancer
RAI: postoperative radioactive iodine
